# AAV-mediated transcription factor EB (TFEB) gene delivery ameliorates muscle pathology and function in the murine model of Pompe Disease

**DOI:** 10.1038/s41598-017-15352-2

**Published:** 2017-11-08

**Authors:** Francesca Gatto, Barbara Rossi, Antonietta Tarallo, Elena Polishchuk, Roman Polishchuk, Alessandra Carrella, Edoardo Nusco, Filomena Grazia Alvino, Francesca Iacobellis, Elvira De Leonibus, Alberto Auricchio, Graciana Diez-Roux, Andrea Ballabio, Giancarlo Parenti

**Affiliations:** 1 Telethon Institute of Genetics and Medicine, Pozzuoli, Italy; 2 Institute of Genetics and Biophysics, CNR, Naples, Italy; 3 Department of Radiology, Second University of Naples, Naples, Italy; 4 Department of Translational Medical Sciences, Federico II University, Naples, Italy; 5 Department of Molecular and Human Genetics, Baylor College of Medicine, Houston, Texas USA; 6 Jan and Dan Duncan Neurological Research Institute, Texas Children’s Hospital, Houston, Texas USA

## Abstract

Pompe disease (PD) is a metabolic myopathy due to acid alpha-glucosidase deficiency and characterized by extensive glycogen storage and impaired autophagy. We previously showed that modulation of autophagy and lysosomal exocytosis by overexpression of the transcription factor EB (*TFEB*) gene was effective in improving muscle pathology in PD mice injected intramuscularly with an AAV-TFEB vector. Here we have evaluated the effects of TFEB systemic delivery on muscle pathology and on functional performance, a primary measure of efficacy in a disorder like PD. We treated 1-month-old PD mice with an AAV2.9-MCK-TFEB vector. An animal cohort was analyzed at 3 months for muscle and heart pathology. A second cohort was followed at different timepoints for functional analysis. In muscles from TFEB-treated mice we observed reduced PAS staining and improved ultrastructure, with reduced number and increased translucency of lysosomes, while total glycogen content remained unchanged. We also observed statistically significant improvements in rotarod performance in treated animals compared to AAV2.9-MCK-eGFP-treated mice at 5 and 8 months. Cardiac echography showed significant reduction in left-ventricular diameters. These results show that *TFEB* overexpression and modulation of autophagy result in improvements of muscle pathology and of functional performance in the PD murine model, with delayed disease progression.

## Introduction

Pompe disease (OMIM 232300) is caused by mutations of the GAA gene and by deficiency of acid alpha-glucosidase (GAA, acid maltase), a lysosomal hydrolase involved in the breakdown of glycogen^1^. Lack of functional GAA results in the typical pathological hallmarks of the disease: i. extensive and generalized intra-lysosomal glycogen storage; ii. massive accumulation of autophagic vesicles and autophagic debris in muscles^2^. The accumulation of autophagic material, likely caused by defective autophagosomal–lysosomal fusions^3,4^, is thought to play a major role in the functional impairment of muscles, and to affect the efficacy of therapies.

Pompe disease is characterized by broad phenotypic variability. Classical infantile-onset patients present within the first months of life with hypotonia, hypertrophic cardiomyopathy, recurrent respiratory infections, and, if untreated, die within the first year of life. In non-classical infantile-onset and in late-onset patients clinical manifestations are mostly related to skeletal muscle involvement with minimal or absent cardiac disease. In all patients the disease is highly debilitating and results in progressive motor handicap, respiratory failure, and premature death.

At present the only approved treatment for Pompe disease, is enzyme replacement therapy (ERT) with human recombinant GAA. Albeit effective in reversing cardiac involvement and prolonging survival in classic infantile-onset patients^5–7^, and in improving or stabilizing neuromuscular deficits and respiratory function in late-onset patients^8,9^, ERT showed limitations. Some patients respond poorly to treatment with transient functional improvements^10,11^. Insufficient response to ERT is likely due to a number of factors, such as disturbed autophagic pathway, age at start of therapy, cross-reactive material (CRIM) status of patients, limited tissue bioavailability of rhGAA, and selective resistance of specific muscles or fiber types to treatment^12^.

The limitations of current therapy stimulated research on second-generation drugs and innovative therapeutic approaches. Novel enzyme preparations with improved muscle targeting properties^13,14^ and small-molecule “chaperones”^15–17^ are in clinical development. Gene therapy with *GAA* gene delivery mediated by different viral vectors and based on different strategies is currently being explored^18–22^, but its clinical translation is not straightforward, with problems in common with ERT, such as the need for transducing many different muscles (heart, skeletal muscles) that account for about 40% of the body mass, the refractoriness to therapies of muscles affected by advanced-stage pathology, and the need to evade immune response to vectors and GAA^23^. The first phase II/III clinical trial based on AAV-mediated GAA gene delivery has been performed by direct intra-diaphragmatic injection in five Pompe patients, with some improvements in ventilatory function^24^. Overall, none of the therapies so far explored or in preclinical and clinical development appears to have the potential to meet all Pompe disease medical needs.

Recent studies have identified Transcription Factor EB (TFEB) as a potential therapeutic target in lysosomal storage diseases in general, including Pompe disease. TFEB is a master regulator of lysosomal biogenesis and autophagy that under basal conditions in most cell types is located in the cytoplasm. TFEB activation and nuclear translocation result in induction of autophagosome formation, improved autophagosome/lysosomes fusions^25^, accelerated recycling of cellular components, stimulation of lysosomal exocytosis and release of lysosomal content into the extracellular space^26–28^. These effects appear particularly attractive to tackle the typical impairment of the autophagic flux observed in Pompe disease muscles.

In a previous study^29^ we showed that overexpression of TFEB in cultured myoblasts from a Pompe disease murine model reduced glycogen stores and lysosomal size, facilitated autophagosome processing, and alleviated excessive accumulation of autophagic vacuoles. Intramuscular delivery of an AAV2/1-TFEB vector resulted in enhanced glycogen clearance and improved muscle architecture. In that study, however, direct intramuscular injection of AAV2/1-TFEB only targeted a single muscle and did not allow for evaluation of functional effects of TFEB overexpression that, in a disorder like Pompe disease, are major and essential measures of muscle involvement. Here, we evaluated the effects of AAV2/9-mediated systemic delivery of the human *TFEB* (hTFEB) gene under the control of the muscle creatine kinase (MCK) promoter on muscle and heart pathology, and we analyzed the impact of the treatment on motor performance and progression of muscle weakness.

## Results

Two different cohorts of knock-out animals were treated with AAV2/9-MCK-hTFEB vectors (referred to as TFEB-treated mice, throughout the text). Two additional groups of knock-out animals injected with equivalent doses of AAV2/9-MCK-eGFP vectors were used as mock knock-out controls (referred to as eGFP-treated mice). A short-term study was intended to explore the effects of TFEB overexpression in the tissues, skeletal muscles and heart, that are most relevant for Pompe disease pathology. A longitudinal study was aimed at exploring at different time-points (3, 5, 8 months of age) the effects of TFEB overexpression on rotarod performance and heart function.

Pompe disease animals were treated with 1.5 × 10^14^ vg/Kg AAV2/9 MCK-hTFEB that in preliminary experiments had been identified as sufficient to obtain detectable infection of target tissues. With this dose the infection efficiency was variable in different target tissues, with the highest levels in liver and heart (Fig. S1). Both in the short-term and longitudinal experiments the levels of infection were comparable and reproducible.

### Effects of TFEB on tissue morphology

The general morphology of muscles and heart was not affected by TFEB treatment. By hematoxylin-eosin staining the tissue architecture of TFEB-treated mice was conserved and comparable with that of wild-type and eGFP-treated animals. Figure S2 shows gastrocnemius and heart architecture.

**Figure 1:**
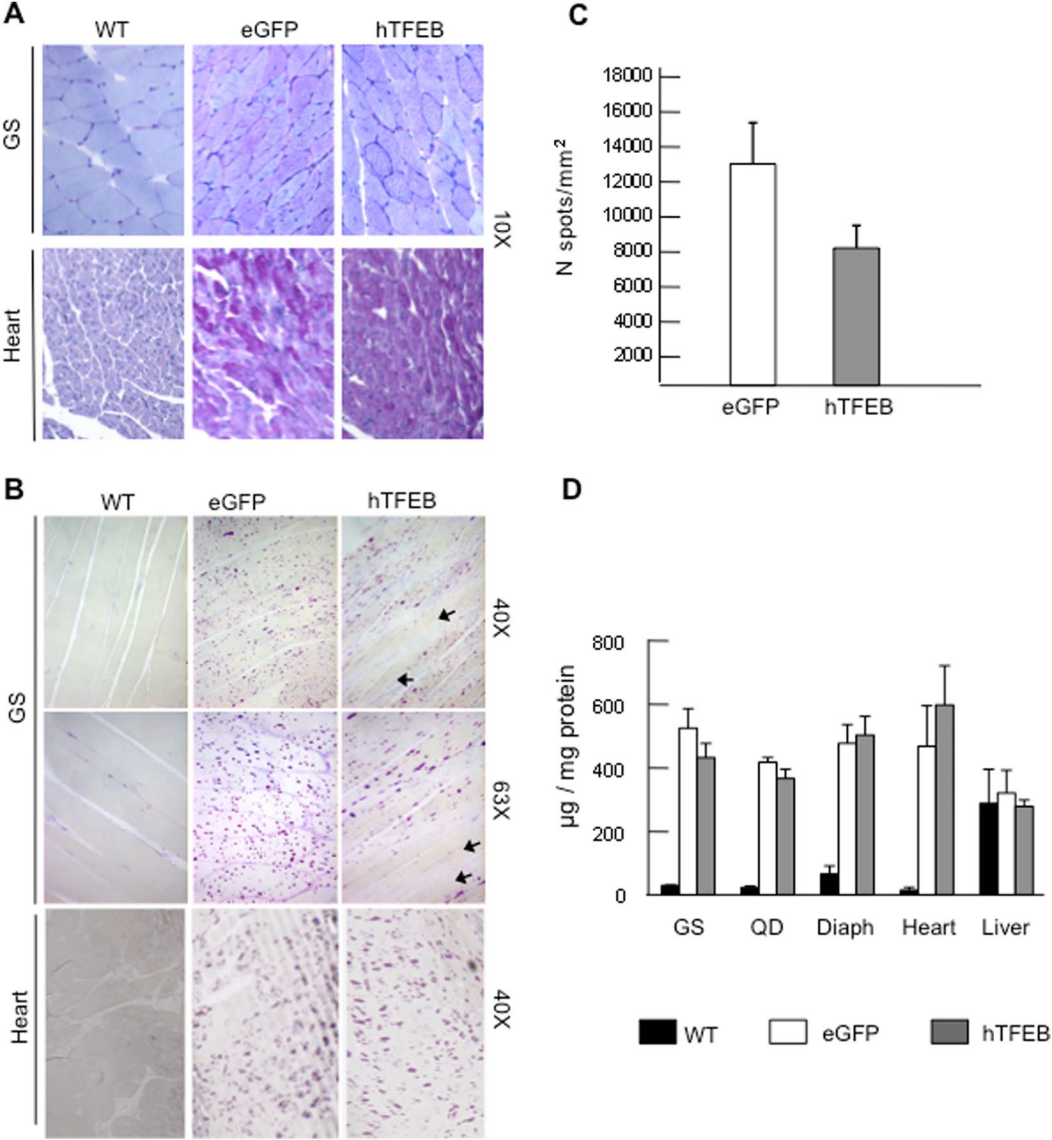
PAS staining in gastrocnemius and heart. PAS positivity has been evaluated in thick 8 µm sections (**A**) from gastrocnemius and heart in 3-mo old mice. In both tissues, knock out eGFP-treated animals showed an increased signal dispersed throughout the cytosol of the fibers compared to wild type (WT) mice; TFEB treatment did not result in significant changes in the staining pattern. Original magnification: 20X. In thinner sections, 1 µm, (**B**) PAS-positive glycogen-filled lysosomes were easily detectable both in gastrocnemius and heart. In TFEB-treated muscles individual fibers (indicated by arrows) showed very few PAS-positive spots. Original magnification: 63X. Scans of Pas stained whole muscle sections form two eGFP-treated and two TFEB-treated knock out animals were acquired using a Hamamatsu Scanner and analyzed by Visiopharm Image analysis software (VIS version 2017.5). The analysis showed decreased density of Pas-positive spots in TFEB-treated animals (**C**). Glycogen content (**D**) was evaluated in gastrocnemius (GS), quadriceps (QD), diaphragm (Diaph), heart and liver of wild type (WT), Pompe eGFP-treated and TFEB-treated 3-mo old mice. The one-tail Student’s t test was performed and no statistically significant differences between the two groups.

We analyzed muscle and heart morphology in 8 μm PAS-stained sections (Fig. 1A). Sections from gastrocnemius revealed an increased background signal (more evident in some cells) in muscle fibers from eGFP-treated Pompe mice compared to wild-type animals, a feature of Pompe disease pathology already reported in humans, and in the murine and canine models of the disease^30–32^. In addition, sparse punctate PAS-positivity was also detectable. TFEB treatment did not result in substantial changes in the staining pattern. Similar results were obtained in heart. Fibers from eGFP-treated mice were intensely stained with PAS, compared to samples from wild-type animals. No significant changes were seen in samples from TFEB-treated mice.

We also performed a PAS staining in thin, 1-µm sections, that allow for better definition of muscle morphology and precise identification of glycogen-filled lysosomal structures (Fig. 1B). In gastrocnemii preparations the cytosolic PAS-positivity was barely detectable in Pompe fibers, while it was possible to clearly identify the spotted, punctate signal consistent with lysosomal-autophagosomal localization of glycogen. Some fibers (indicated by arrows at both 40x and 63x magnifications) were almost devoid of PAS-positive spots. Image analysis of scans of whole muscles sections showed that in TFEB-treated animals the average density of PAS-positive spots was reduced (8,144.9 per mm^2^) compared to eGFP-treated animals (13,189.3 per mm^2^) (Fig. 1C). The technical procedures for resin inclusion and PAS-staining, however, did not allow for identification of transduced cells or fiber type in these sample preparations.

In heart both thick 8 µm and 1-µm section showed the presence of generalized PAS-positive punctate staining), without apparent reduction in the number (and intensity of staining) of puncta between TFEB-treated and eGFP-treated animals.

A biochemical analysis of glycogen showed highly increased levels in eGFP-treated knock-out animals compared to wild-type in all tissues examined with the exception of liver (Fig. 1D). In liver, where cytosolic glycogen stores are predominant and actively participate in the regulation of glucose homeostasis, we found relatively high levels of glycogen also in wild-type mice and no significant differences between wild-type and Pompe mice (both  eGFP-treated and TFEB-treated), indicating that in liver the lysosomal fraction of total glycogen is negligible compared to cytosolic glycogen. In all tissues examined TFEB treatment did not cause significant decrease in total glycogen levels.

**Figure 2:**
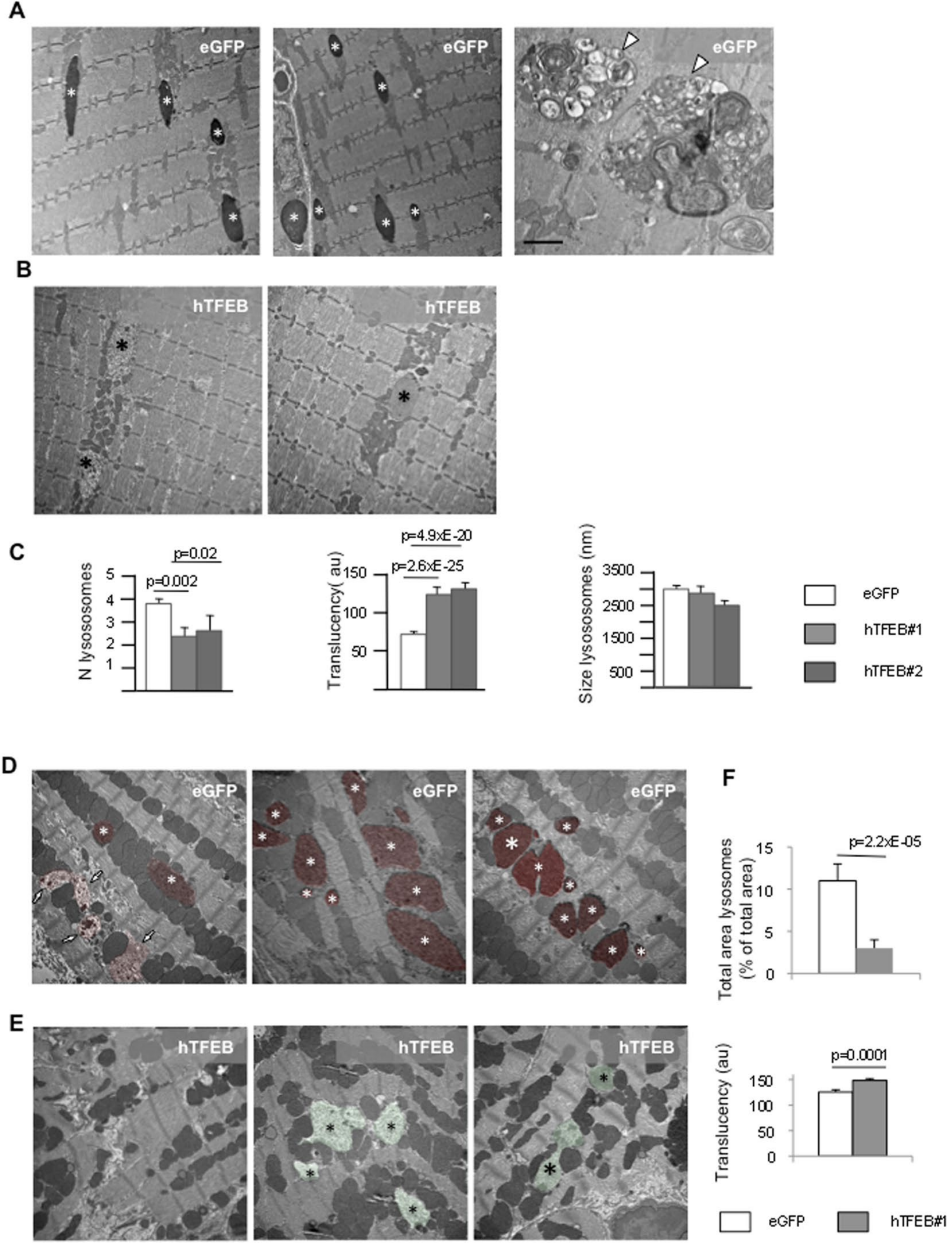
Ultrastructural analysis of gastrocnemius and heart. (**A**) Gastrocnemii from eGFP-treated animals showed the hallmarks of the disease, typically glycogen-filled lysosomes, appearing as densely packed particles (left and center panels, white asterisks; 8000x), and large areas of accumulation of autophagic material containing autophagosomes, multivesicular bodies and multimembrane structures (right, white arrowheads; scale bar 800 nm). (**B**) In TFEB-treated mice less lysosomes were found and their content differed significantly respect to control group. The majority of those lysosomes exhibited higher translucency, indicating reduced density of glycogen stores (black asterisks; 8000x). (**C**) Morphometric analysis. In TFEB-treated mice the number of glycogen-containing lysosomes was significantly reduced (p = 0.002 and p = 0.02, respectively), compared to samples from eGFP-treated animals. The translucency of lysosomes was also highly and significantly increased (p = 2.6XE-25 and p = 4.9xE-20, respectively). The size of lysosomes did not show significant changes. (**D**) In hearts from knock-out eGFP-treated animals a large number of glycogen-filled lysosomes were observed (red-colored structures 9000X). (**E**) large areas without glycogen-containing lysosomes were easily observed in TFEB-treated animals (left; 9000X). (**F**) Morphometric analysis showed reduced total area of lysosomes in hearts from TFEB-treated mice and increased translucency of lysosomes.

To study the ultrastructural effects of TFEB overexpression in TFEB-treated mice, we performed an electron microscopy (EM) analysis in gastrocnemii and heart. Gastrocnemii from  eGFP-treated animals showed the hallmarks of the disease, typically glycogen-filled lysosomes, appearing as densely packed particles (Fig. 2A, **left and center**, **white asterisks**; **8000X magnification**), and large areas of accumulation of autophagic material containing autophagosomes, multivesicular bodies and multimembrane structures (Fig. 2A, **right**, white arrowheads). All these features were absent in wild type animals (not shown). In TFEB-treated mice less lysosomes were found and their content differed significantly with respect to control eGFP-treated group. The majority of those lysosomes exhibited higher translucency, indicating reduced density of glycogen stores (Fig. 2B, black asterisks). In TFEB-treated mice the number of glycogen-containing lysosomes was significantly reduced (p = 0.002 and p = 0.02, respectively), compared to samples from eGFP-treated animals (Fig. 2C). The translucency of lysosomes was also highly and significantly increased (p = 2.6XE-25 and p = 4.9xE-20, respectively). The size of lysosomes did not show significant changes.

In heart preparations from control eGFP-treated knock-out animals a large number of glycogen-filled lysosomes were observed (Fig. 2D; **9000X magnification**), while large areas without glycogen-containing lysosomes were easily observed in TFEB-treated animals (Fig. 2E, **left**; **9000X magnification**). Interestingly, both fully (red colored-structures and white asterisks), and partially glycogen-filled lysosomes (red color and white arrows) were found in eGFP-treated mice. However, the majority of these structures exhibited high electron density content due to high glycogen content (see white asterisks in all images Fig. 2D). In tissues from TFEB-treated mice two important features were detectable. Glycogen inclusions occupied a reduced area (green color and black asterisks in panel E) compared to eGFP-treated animals (panel D, red color and white asterisks). Тhis result was confirmed by morphometric analysis (Fig. 2F, total area lysosomes). Second, in contrast to eGFP-treated knock-out tissues, the majority of lysosomes in TFEB-treated animals (Fig. 2F, translucency) indicated by green color and black asterisks in panel F) contained less glycogen indicating reduced glycogen stores.

### Autophagy and atrophy-related markers

The abnormalities of the autophagic compartment are an important feature in Pompe disease. These abnormalities have been considered as major players in the pathology of skeletal muscles, and there is substantial evidence supporting the idea that the autophagic build-up is involved in mistrafficking and impaired efficacy of the recombinant enzymes used for enzyme replacement therapy^29,33^.

We analyzed by western-blot the isoforms of LC3, a known and widely used marker of autophagy^34^ and p62/SQSTM1, a marker of autophagic degradation of poly-ubiquitinated substrates^34^ in samples from gastrocnemii and hearts obtained from wild type, eGFP-treated knock-out and knock-out TFEB-treated animals.

**Figure 3:**
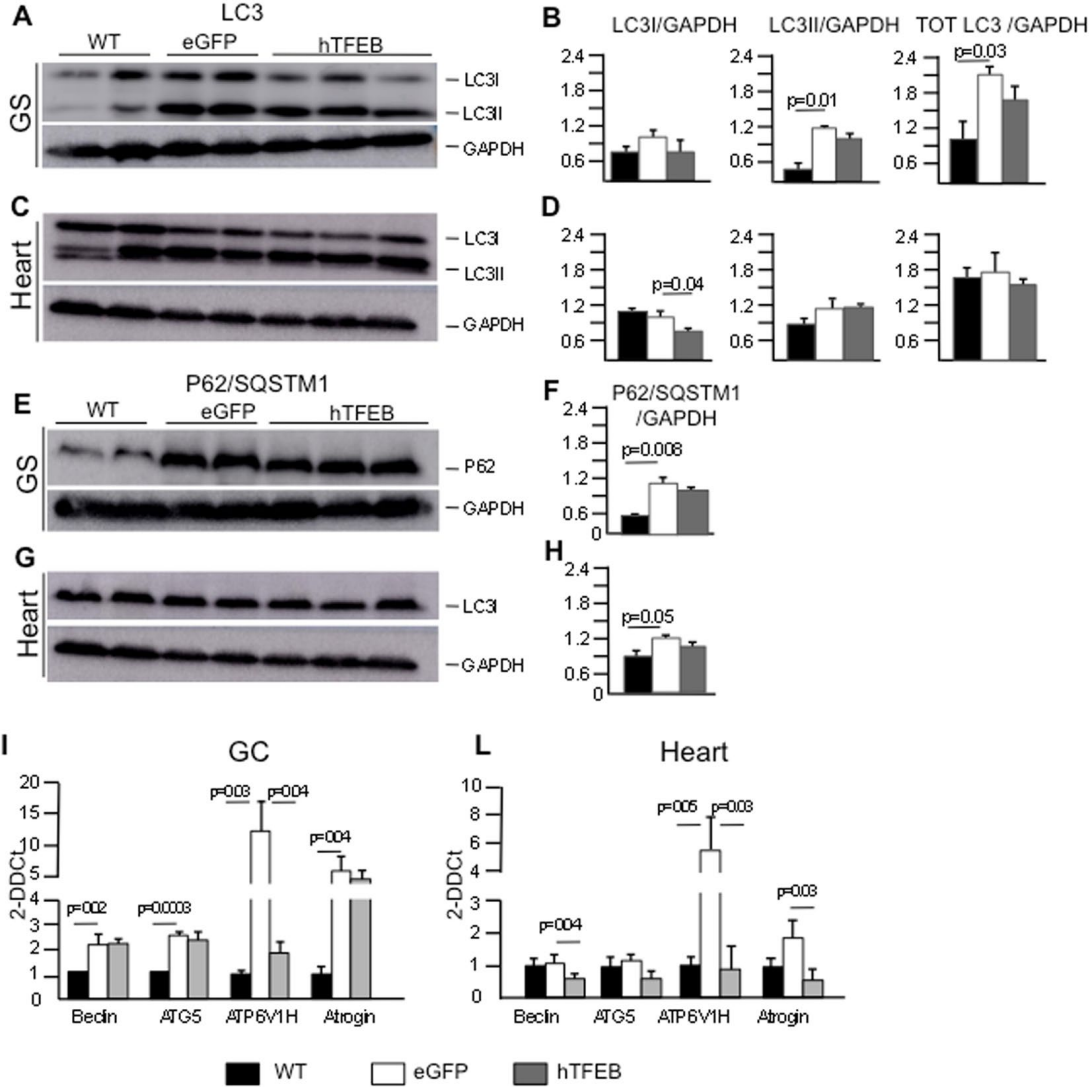
Evaluation of autophagy-related and atrophy markers. Western blot analysis of LC3I/II (**A**–**D**) and P62/SQSTM1 (**E**–**H**) in muscles (gastrocnemius and heart) from 3-mo-old WT, eGFP-treated and TFEB treated mice. GAPDH was used as a loading control. The blots were cropped eliminating, for clarity, lanes that were not relevant for the results shown; in the blots contrast was enhanced electronically (and not overexposed). Full-length blots are presented in Supplementary Fig. S3. Graphical representation of relative intensity data are shown in the graph as mean ± SEM. mRNA transcript abundance of autophagy-related and atrophy genes was assessed by RT-PCR (**I**–**L**). Values are represented as RNA fold change and show means ± SEM. The data are analyzed using one-tailed Student’s t-test.

Total LC3, LC3II (Fig. 3A–D) and p62/SQSTM1 (Fig. 3E–H) levels were significantly increased in gastrocnemius extracts from eGFP-treated knock-out animals compared to wild-type. These findings are consistent with the results of previous studies^2,3^ and with the known impairment of autophagy in Pompe disease skeletal muscles. In heart only p62/SQSTM1 was significantly increased.

In gastrocnemii from TFEB-treated animals LC3I, LC3II, total LC3 and p62/SQSTM1 levels showed a trend towards decrease, although not statistically significant. LC3I showed a significant decrease in hearts from TFEB-treated animals.

We also analyzed by RT-PCR the expression levels of genes involved in different steps of the autophagic pathway, including Beclin-1, a mammalian ortholog of yeast Atg6 that plays a central role in regulating autophagy through interactions with Vps34^35^, ATG5, a ubiquitin ligase involved in autophagosome elongation and LC3I lipidation^34^, and ATP6V1H, a component of the amino acid-sensing machinery involved in the activation of mTORC1^36,37^ and a factor required for lysosome/autophagosome fusions^38^. All three genes showed significant differences between wild-type and Pompe disease eGFP-treated animals (Fig. 3I–L). TFEB treatment resulted in a significant decrease (that is towards wild-type values) in the expression of ATP6V1H (p = 0.04). In heart both Beclin-1 and ATP6VH1 showed significant decreases (p = 0.04 and p = 0.03, respectively).

Other genes encoding factors involved in the autophagic pathway (PGC-1α, ATG14, Mucolipin1, Catepsin F and Catepsin L) did not show differences between wild-type and eGFP-treated knock-out animals and therefore were not considered informative on the effects of TFEB treatment (data not shown).

The activation of the pathway of atrophy is a common finding in muscles from Pompe disease patients^3,39^. We analyzed the expression levels of Atrogin1, an intermediate in this pathway that has been shown to be up-regulated in Pompe disease^39^. In Pompe eGFP-treated mice the expression of Atrogin1 in gastrocnemius and heart was increased, compared to wild-type animals (Fig. 3I–L). TFEB treatment resulted in decreased expression of Atrogin1, that reached statistical significance in heart (p = 0.02).

### Behavior and functional studies

The longitudinal study was aimed at evaluating the functional effects of TFEB overexpression. The performance of TFEB-treated animals (n = 6, males) was compared to that of wild-type mice (n = 13, males) and knock-out eGFP-treated animals (n = 9, males). The mice were examined at different timepoints for behavior according to tests that have been previously used in similar studies to characterize the *Gaa* knock-out phenotype and in preclinical *in vivo* studies on the effects of therapies for Pompe disease.

**Figure 4:**
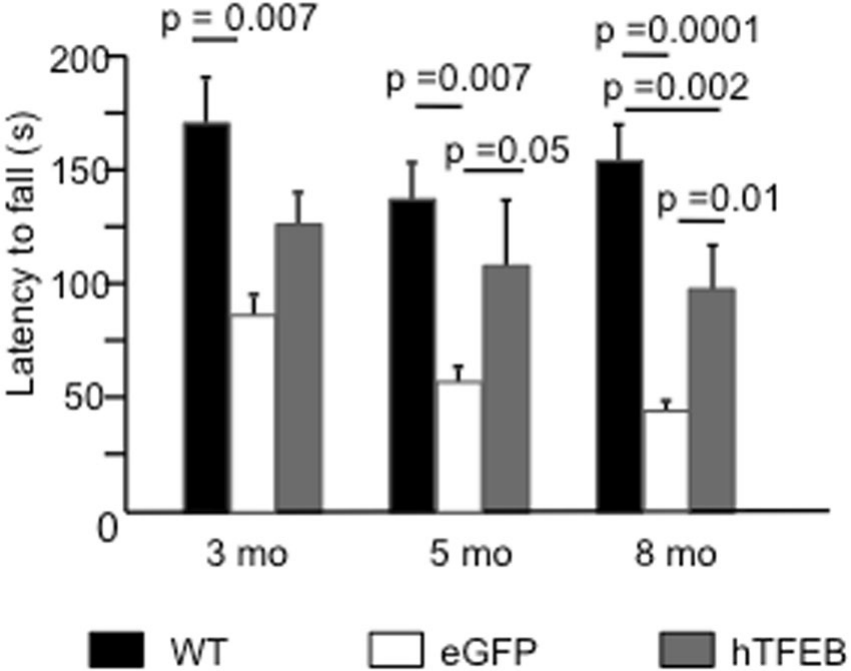
Rotarod tests. The functional effect of TFEB overexpression was evaluated on the performance of wild type (WT), eGFP-treated and TFEB-treated knock out male mice at different timepoints (3, 5 and 8 months) by the rotarod test. Significant differences in performance between WT and eGFP-treated mice since 3 months, progressively increasing during time. TFEB-treated animals performed significantly better than untreated mice at 5 and 8 months. P value is calculated with One-way ANOVA.

Rotarod explores aerobic motor function, endurance, cardiac and respiratory function, and coordination and has been the most commonly used test in previous studies in the Pompe disease mouse model. Significant differences in rotarod performance between wild-type and knock-out eGFP-treated animals were already seen at 3 months (One-way ANOVA, F2/25 = 6.023, *p* = 0.007) (Fig. 4) and progressively became more evident during follow-up. Also TFEB-treated animals showed significant differences compared to wild-type mice, but at 3 months statistical significance was lost, indicating an initial beneficial effect of treatment. At 5 (p = 0.007) and 8 (p = 0.0001) months TFEB-treated animals performed significantly better than eGFP-treated knock-out mice. These results indicate a significant and substantial delay in the progression of motor impairment in Pompe mice.

**Figure 5:**
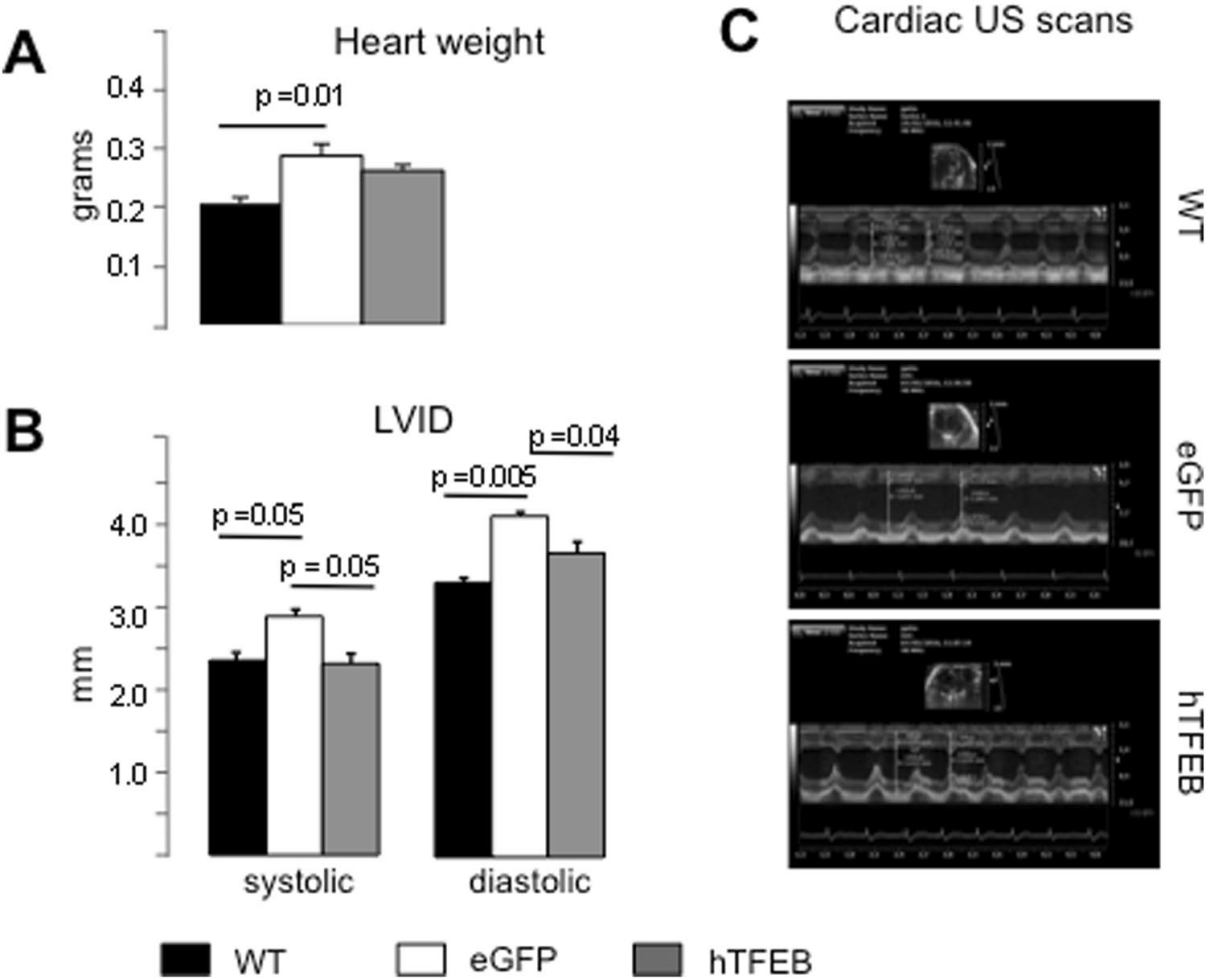
Cardiac parameters. (**A**) Heart weight expressed in grams of 8-mo old wildtype, knock out eGFP-treated and TFEB-treated mice. P value is obtained using one-tailed Student’s t-test. (**B**) Left Ventricular Internal Diameter. In TFEB-treated animals both systolic and diastolic diameters were significantly decreased compared to eGFP-treated knock out animals. P value is obtained using one-tailed Student’s t-test. (**C**) Representative echocardiography of wild type (WT), eGFP-treated, and TFEB treated knock out male mice at 8-mo old.

Cardiomyopathy is one of the clinical manifestations observed in the Pompe disease mouse model^30,40^, although not as severe as that seen in classical infantile-onset patients. On inspection, all knock-out animals (both eGFP-treated and TFEB-treated) showed larger hearts than controls, with significantly increased cardiac weight (Fig. 5A). In TFEB treated animals heart weight was lower, although not statistically significant.

At the end of the study we performed cardiac echography in 3 TFEB-treated mice, and in 3 wild-type and 3 eGFP-treated knock-out mice for comparison. We found significantly increased systolic and diastolic left ventricular diameters in eGFP-treated animals compared to wild-type (Fig. 5B). In TFEB-treated animals both diameters were significantly decreased (p = 0.05 and p = 0.04, respectively) compared to eGFP-treated mice. No significant changes were seen in septum and posterior wall thickness (not shown). Figure 5C shows representative echographies from wild-type, knock-out eGFP-treated, and TFEB-treated mice.

## Discussion

In this study we have tested the effects of systemic AAV2/9-mediated TFEB gene therapy in the murine model of Pompe disease. TFEB has known effects on the autophagic pathway, impaired in Pompe disease patients and animal muscles, and on lysosomal exocytosis, that is expected to activate an alternative route for disposal of glycogen stores. Modulation of autophagy (possibly improving the autophagic flux) and reduction of the stored substrate has the potential to attenuate muscle pathology and translate into beneficial functional effects.

For TFEB gene delivery we chose an AAV2/9 serotype that has been reported to have a good profile in terms of biodistribution and expression in skeletal muscles and heart when administered systemically to fetal and neonatal mice or to late-gestation primates^41–43^. In preliminary studies we evaluated the infection efficiency of different vector doses, and we selected the dose of 1.5 × 10^14^ vg/Kg that appeared to achieve sufficient levels of tissue transduction, and to obtain detectable functional effects. Similar doses have been used for preclinical studies on correction of Duchenne muscular dystrophy in dogs^44^. Like in other studies based on this approach, heart and liver were efficiently infected, with lower levels of infection in skeletal muscles. Similar biodistribution was also observed with other strategies. ERT-treated animals and patients show good uptake of rhGAA and clinical response in heart, but poor uptake of the recombinant enzyme in skeletal muscles^45–47^. Transduction of many different muscles with viral vectors and correction of pathology still remain major challenges for several muscular disorders (including muscular dystrophies), and require elevated vector titers to obtain efficient and widespread transduction^18–23^. Studies are in progress to obtain pharmacological enhancement of muscle transduction in the Pompe disease murine model^19^ and may lead in the future to optimization of viral doses.

TFEB treatment resulted in attenuation of muscle pathology, both by PAS staining and EM analysis, in modulation of the expression of genes involved in the autophagic pathway and atrophy, and in motor and cardiac functional improvements. These improvements appear to be comparable to those obtained in the majority of patients under ERT in terms of rescue of pathology and performance^47,48^.

The finding of unchanged glycogen levels in tissue homogenates from TFEB-treated mice in comparison with knock-out animals was intriguing and apparently in contrast with our previous study^29^ based on direct injections in a single muscle of an AAV-TFEB vector. In that study, however we injected 10^11^ vp in three different sites, resulting in high levels of TFEB expression. In the present study we could not reach high expression levels, as suggested by the data of TFEB infection and the results of PAS staining. In addition, the biochemical assay in tissue homogenates is not able to discriminate between cytosolic and lysosomal glycogen. A secondary (and non-specific) accumulation of cytosolic glycogen has been observed in muscles from Pompe disease animal models^30,32,48^, and in general in muscular dystrophies, and must be taken into careful consideration in the differential diagnosis of myopathies. In addition, recent work in the mouse model of the disease has demonstrated a robust increase in factors that promote glycogen synthesis (such as GLUT4 and glycogen synthase) and dysregulation of muscle phosphorylase, both factors potentially contributing to secondary accumulation of cytosolic glycogen^49^. Another recent study points out that TFEB has also a metabolic effect resulting in increased muscle glycogen in TFEB-overexpressing transgenic mice that may contribute to masking the effects on lysosomal glycogen clearance^50^. These metabolic effects of TFEB, in combination with the low levels of infection, may explain the lack of changes in glycogen levels in tissues from treated animals.

Although it was not possible to show a clear effect of TFEB treatment on the markers of autophagy studied, overall, the trend was towards normalization, or to reach levels that were closer to those observed in control mice, which may indicate a modulating effect on the autophagic pathway. One of the markers, ATP6VH1 that is involved in the acidification of lysosomes and has recently emerged as a key player in the modulation of the mTOR pathway^38,51^ showed substantial and significant changes in knock-out mice vs WT, and in eGFP-treated vs TFEB-treated  mice. These results point to a modulatory effect of TFEB on the autophagic flux. This effect may prove of particular interest considering that dysregulation of autophagy has been shown to impact on muscle function and on enzyme replacement efficacy^29,33^. Even a partial rescue of autophagy may improve the uptake of either recombinant GAA, in patients undergoing enzyme replacement therapy, or circulating GAA, in patients treated by liver-directed gene therapy. The combination of TFEB and other approaches aimed at enzymatic correction should be evaluated in future preclinical studies. A possible role of TFEB therapy to enhance substrate clearance in advanced stages of disease should also be explored.

An important and novel finding was the effect of AAV-TFEB systemic delivery on functional performance and disease progression. This finding adds critical information to our previous study based on direct intra-muscular injection of an AAV-TFEB-vector^29^. Improvements in rotarod performance, that explores aerobic muscle function, physical performance and cardiac function, were statistically significant at 5 and 8 months. In addition, we found improved cardiac parameters in TFEB-treated animals, with decreased left-ventricular systolic and diastolic diameters, that may contribute to the improvements in rotarod performance. Overall, the results of our functional tests indicate substantial delay in the progression of the disease, a major therapeutic goal in the treatment of lysosomal storage diseases, including Pompe disease^11,46,52,53^ and suggest that future treatments directed towards modulation of secondarily disregulated pathways in Pompe disease may prove beneficial as adjunct therapies.

## Materials and Methods

### Constructs and AAV2/9 vectors

Two different constructs were generated for AAV2/9 vector preparations. Starting from the backbone plasmid pAAV2.1, the sequence of the muscle-specific promoter MCK MCK (Muscle Creatine Kinase) and the cDNA encoding the therapeutic hTFEB (human transcription factor EB) protein and the control eGFP (enhanced Green Fluorescent Protein) were subcloned into pAAV2.1 by digestion with restriction enzymes. The 3x-flag tag was synthesized as a primer and after digestion was cohesively ligated to the pAAV2.1MCK-hTFEB backbone vector. The final construct was named pMCK-hTFEB-3Xflag. The vectors were then packaged into AAV9 capsids and were produced by triple transfection of HEK293 cells followed by two rounds of CsCl_2_ purification according to published procedures. Vector titers were determined by DNA dot blot at ~1 × 10^13^ viral genome particles per ml in 1X PBS plus 5% glycerol^54^.

### Animals and study design

Two different cohorts of animals were treated with AAV2/9-MCK-hTFEB vectors. Two additional groups of animals injected with equivalent doses of AAV2/9-MCK-eGFP vectors were used as mock knock-out controls (referred to as eGFP-treated in the text). For systemic studies, the AAV2/9 vectors (1,5 × 10^14^ vg/Kg) were suspended in saline and delivered (range volume 125 µL–390 µL) into 1-mo old mice through retro-orbital sinus injection.

For the short-term study (TFEB-treated animals N = 4; eGFP-treated N = 3) gastrocnemius and heart samples were collected at the age of 3 months for tissue pathology, viral genome copies, glycogen content, and analysis of markers of autophagy. Genome copies analysis and glycogen assay were also performed in samples from quadriceps, diaphragm, and liver. The animals were sacrificed under anesthesia by cervical dislocation, after 6 hours of starvation.

For the longitudinal study (TFEB-treated animals N = 6; eGFP-treated N = 9; wild-type N = 13) behavioral analysis was performed in groups of animals at different time-points (3, 5, 8 months of age). At the end of the study, after the last functional evaluation and heart ultrasound scan analysis, the animals were sacrificed and samples for genome copy analysis were collected.

### Viral Genome Copies and Gene Expression analyses

Absolute quantification by real-time PCR was used to measure the distribution of AAV viral genomes in tissues from Pompe mice. Viral genome copies were evaluated in gastrocnemius, quadriceps and diaphragm, liver and heart. Tissues were harvested in a manner that prevented cross-contamination, snap frozen in liquid nitrogen and stored at −80 °C until genomic DNA (gDNA) was extracted. gDNA was isolated using a DNeasy blood and tissue kit (Qiagen, Hilden, Germany) according to the manufacturer’s instructions. AAV genome copies were quantified by real-time PCR on 100 ng of gDNA using a set of primers/probe specific for the viral genome and TaqMan universal PCR master mix (Applied Biosystems, Foster City, CA). A standard curve was performed using plasmid DNA containing the same target sequence. The viral genome copies reported were normalized per molecule of diploid genome.

The expression levels of TFEB and of different markers of the autophagic pathway, namely, Beclin 1, ATG5, ATP6V1H, were analyzed by RT-PCR in samples from gastrocnemius and heart. Total RNA was extracted from tissues using RNAeasy mini Kit (Qiagen, Hilden, Germany). 1 µg of RNA was used to generate cDNA with QuantiTect Reverse transcription Kit (Qiagen) and analyzed by quantitative real-time PCR using SYBR Green master mix (Roche, Basel, CH). All data were normalized to GAPDH. The oligonucleotide primers used are shown in Table S1.

All amplifications were run on a Light Cycler 480 Instrument detection system (Roche, Basel, Switzerland).

### Glycogen assay

Glycogen concentration was assayed in tissue lysates by measuring the release of glucose after digestion with *Aspergillus niger* amyloglucosidase (Sigma Aldrich, Saint Louis, MO, USA). Data were expressed as µg of glycogen/mg of protein.

### Histological analyses

Tissue pathology was studied by standard hematoxylin-eosin (H-E) staining, and by the glycogen-specific PAS staining according to the manufacturer’s instructions (Bio-Optica, Milan, IT). For PAS staining tissues harvested and snap frozen in 2-Methylbutane were sectioned using a cryostat, in standard 8µm-thick preparations. In addition to these preparations, samples from gastrocnemius and heart were embedded in resin, sectioned into 1 µm sections, and stained with PAS. One-µm sections of entire muscles were scanned by Leica DM 5500 microscope (Leica, Wetzlar, Germany).

Images of Pas stained thin section for quantification were also acquired using a Hamamatsu Scanner. Automated count of the number of spots in whole muscle sections from two eGFP-treated and two TFEB-treated mice was done with Visiopharm Image analysis software (VIS version 2017.5).

### Electron microscopy

For Electron microscopy (EM) muscle tissues were fixed in 1% glutaraldehyde in 0.2 M HEPES buffer and post- fixed in uranyl acetate and OsO_4_. After dehydration through a graded series of ethanol and propylenoxide, the tissue was embedded in the Epoxy resin (Epon 812, Sigma–Aldrich) and polymerized at 60 °C for 72 h. EM images of thin sections were acquired using a FEI Tecnai-12 electron microscope (FEI, Eindhoven, Netherlands) equipped with a VELETTA CCD digital camera (Soft Imaging Systems GmbH, Munster, Germany).

Quantification of the number of lysosome-like organelles and their dimensions was performed using the same magnification within 141 μm square field of view. Fifty fields for measurement were chosen randomly through the thin sections containing different fibers. The Kolmogorov–Smirnov test was used for comparison of cumulative distributions of lysosomal size in myotubes and muscle fibres; Wilcoxon rank sum test was used for comparison of median values. Student’s t-test was used for all other comparisons. Differences were considered significant at p < 0.05. Clearance of lysosomes from glycogen was measured as translucency of lysosomal structures using Image J software and expressed in arbitrary units (au). In samples from heart morphometric analysis was performed using iTEM software (Olympus SYS, Germany). The percentage of tissue area occupied by glycogen-containing inclusions was calculated as a ratio of area corresponding to glycogen-containing inclusions to entire field of view. Fourty-100 μm square fields per each specimen were analyzed using the same magnification.

### Western Blot analysis

For western blot analysis tissue samples were lysed in lysis buffer with proteinase inhibitor cocktail (Sigma-Aldrich, St Louis, MO, USA), homogenized with TissueLyser (Qiagen, Hilden, Germany) and extracted. Protein concentration was determined by the BCA method (Thermo Scientific, Rockford, IL, USA). 20 µg protein per sample was separated by 10% SDS-PAGE and electrotransferred to PVDF membranes. After blocking, membranes were incubated at 4 °C overnight with the following primary antibodies: an anti-p62 monoclonal antibody (Abnova, Taipei, Taiwan); an anti-murine LC3B rabbit polyclonal antibody (Novus, Littleton, CO, USA) (both at a 1:600 dilution); an anti-mouse GAPDH monoclonal antibody (Santa Cruz Biotechnology, Dallas, TX, USA) (1:12000 dilution). A sheep anti-mouse IgG and a donkey anti rabbit IgGboth conjugated with horseradish peroxidase (GE Healthcare, Little Chalfont, UK) were used as secondary antibodies. Protein bands were visualized with SuperSignalWest Pico Chemiluminescent substrate (Thermo Scientific, Rockford, IL, USA) by exposure to Imager 600 (GE Amersham, Little Chalfont, UK).

### Apparatus and behavioral procedures

Mice were housed in Plexiglas cages (18 × 35 × 12 cm^3^) with free access to food and water and kept at a temperature of 20–23 °C. Tests were carried out in a behavioral testing room maintained under constant light, temperature, and humidity during daylight hours (9 a.m.–6.00 p.m.). Before each behavioral task, animals were acclimatized to the testing room for at least 30 min.

Motor performance and coordination was tested by using a rotarod apparatus for mice (Ugo Basile, Comerio, Italy) with five rods. Briefly, on the first day each mouse was gently placed on each rod set at a steady slow speed of 4 rpm and trained to remain on the rod for 60 sec. After this habituation trial, each animal was exposed to 4 trials per day for 2 consecutive days, with an intertrial interval of 30 min. The trial started when all mice moved in the correct direction. The rotarod was set at increasing speeds ranging from 5 to 40 rpm over 5 min^55^ and the animals were left on the rod for an additional 5 min. The latency to fall off the rod within this period was recorded. The observer was blinded to genotype and treatment.

### Statistics

The unpaired t test was performed using the GraphPad Prism Program, version 6 (San Diego, CA). Values were considered statistically significant at P < 0.05. Power analysis for unbalanced one-way ANOVA Power Analysis of mice cohort size of the longitudinal study resulted in a value of 0.99.

### Cardiac ultrasound

Ultrasound examinations were performed by an expert radiologist with a VisualSonics Vevo 2100 unit, (VisualSonics Inc, Toronto, Canada) for animal research. Each mouse was anesthetized with isoflurane and positioned on a dedicated table in supine position. Heart rate and body temperature were constantly monitored. Heated sonographic gel (Aquasonic 100; Parker Laboratories, Inc, Fairfield, NJ) was applied on the thorax. Images were acquired by using a 18–38 MHz linear array (MS 400). The echocardiographic study was performed in two-dimensional mode (B-mode) in the parasternal long axis and short parasternal axis. With the transducer placed in short parasternal axis, the following end-diastolic and end-systolic parameters were acquired in M-mode with the M-Mode axis placed at the mid level of the left ventricle, just medial of the papillary muscle: interventricular septum thickness; left ventricular diameter; left ventricular posterior wall thickness.

### Study approval

Pompe mice were maintained at the Cardarelli Hospital Animal House (Naples, Italy). All protocols involving animal experiments were conducted in accordance with the guidelines of the Animal Care and Use Committee of Cardarelli Hospital and authorized by Institutional Animal Care and Use Committee (IACUC) guidelines for the care and use of animals in research. The study was approved by the Italian Ministry of Health, IACUC no°523/2015-PR authorized on 06/11/2015.

## Electronic supplementary material

Supplementary Information
