# Supplementary material for: AAV-mediated transcription factor EB (TFEB) gene delivery ameliorates muscle pathology and function in the murine model of Pompe Disease
Source: Sci Rep. 2017 Nov 8;7:15089. doi: 10.1038/s41598-017-15352-2 (PMC5678083; doi:10.1038/s41598-017-15352-2)
Supplement: Supplementary file 1 — Supplementary Information [file 41598_2017_15352_MOESM1_ESM.pdf]

Supplementary information

Title

AAV-mediated transcription factor EB (TFEB) gene delivery ameliorates muscle pathology and function in the murine model of Pompe Disease

Authors and Affiliations

Francesca Gatto[1], Barbara Rossi[1], Antonietta Tarallo[1], Elena Polishchuk[1], Roman Polishchuk[1], Alessandra Carrella[1], Edoardo Nusco[1], Filomena Grazia Alvino[2], Francesca Iacobellis[3], Elvira De Leonibus[1,2], Alberto Auricchio[1,4], Graciana Diez-Roux[1], Andrea Ballabio[1,4,5,6] Giancarlo Parenti[1,4]

[1]Telethon Institute of Genetics and Medicine, Pozzuoli, Italy

[2]Institute of Genetics and Biophysics, CNR, Naples, Italy

[3]Department of Radiology, Second University of Naples, Naples, Italy

[4]Department of Translational Medical Sciences, Federico II University, Naples, Italy

[5]Department of Molecular and Human Genetics, Baylor College of Medicine, Houston, Texas, USA

[6]Jan and Dan Duncan Neurological Research Institute, Texas Children's Hospital, Houston, Texas, USA

Correspondence should be addressed to G.P. (parenti@unina.it)

Giancarlo Parenti

Via Campi Flegrei 34, Pozzuoli, Italy

+39 081 7463390

parenti@tigem.it and parenti@unina.it

FIGURE S1
GC levels in tissues

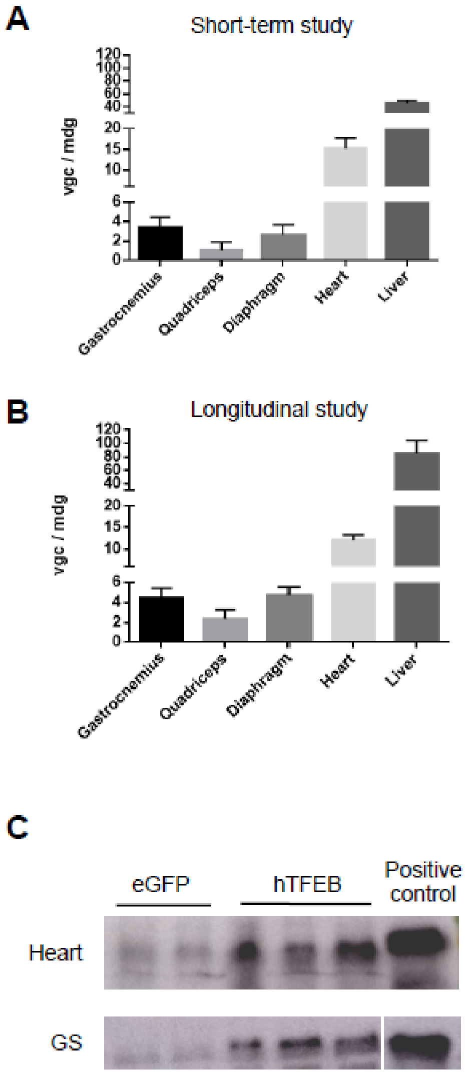

Figure S1. Viral genome copies in tissues. The biodistribution of AAV viral genomes was evaluated by qPCR in short-term (**A**) and longitudinal (**B**) studies in different tissues. TFEB-treated animals are shown in the graph as mean ± SEM. Western blot analysis of 3Xflag (**C**) in muscles from 3-mo-old eGFP and TFEB treated mice. The blots were cropped; full-length blots are presented in Supplementary Figure S4 and S5.

FIGURE S2

H-E staining in muscles

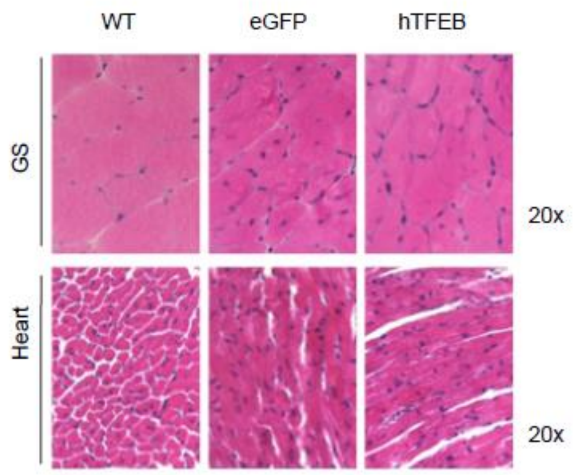

Figure S2. Hematoxylin and Eosin staining. The H&E staining has been evaluated in 10µm snap frozen section sections (**A**) from gastrocnemius and (**B**) heart in 3-mo old mice. In both tissues, TFEB treatment did not result in morphological changes in muscle fibers. Original magnification: 20X.

FIGURE S3

Western blot analysis of autophagy markers (fig 3)

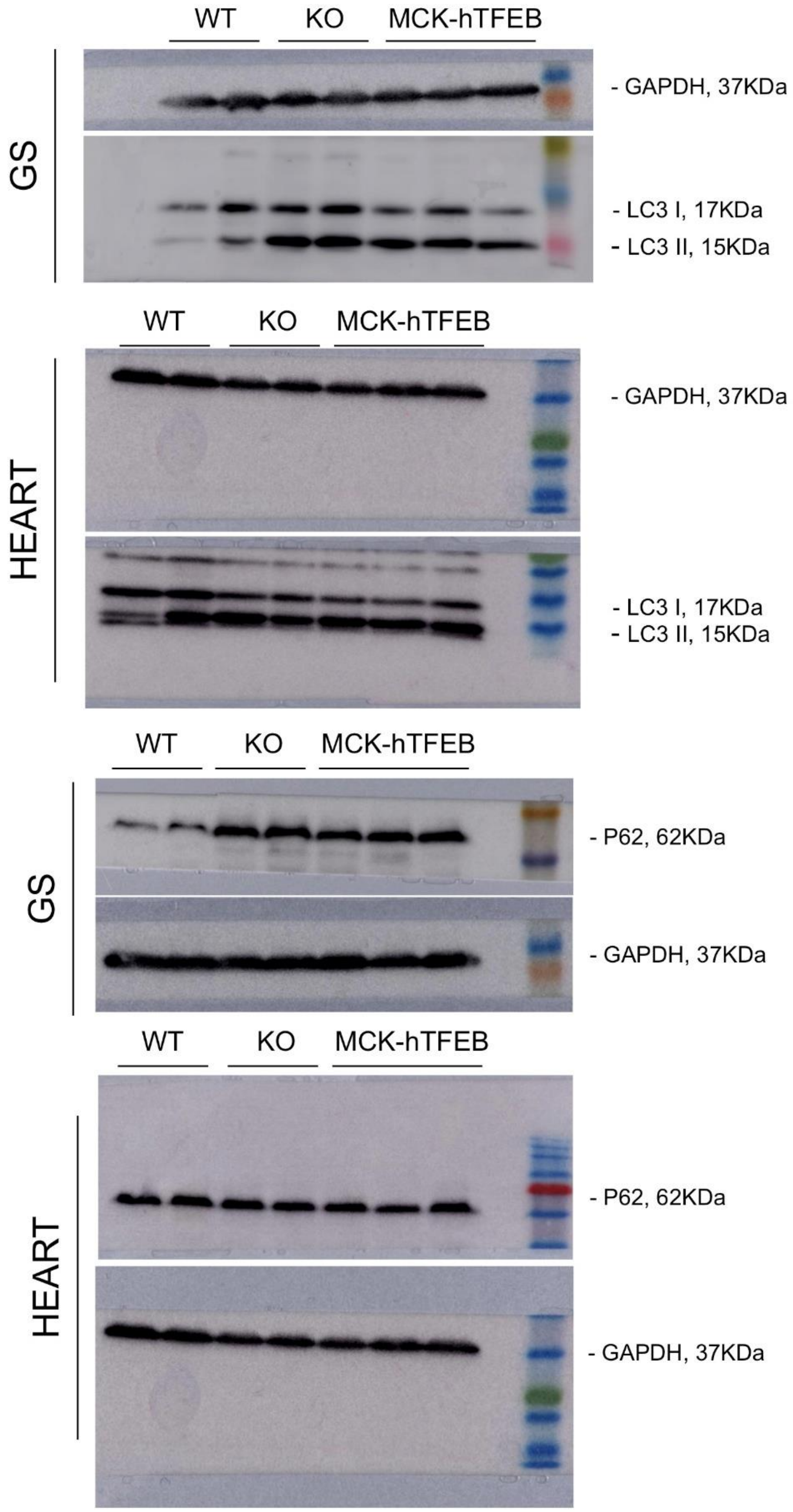

Figure S3. Original blots of autophagic markers.

FIGURE S4

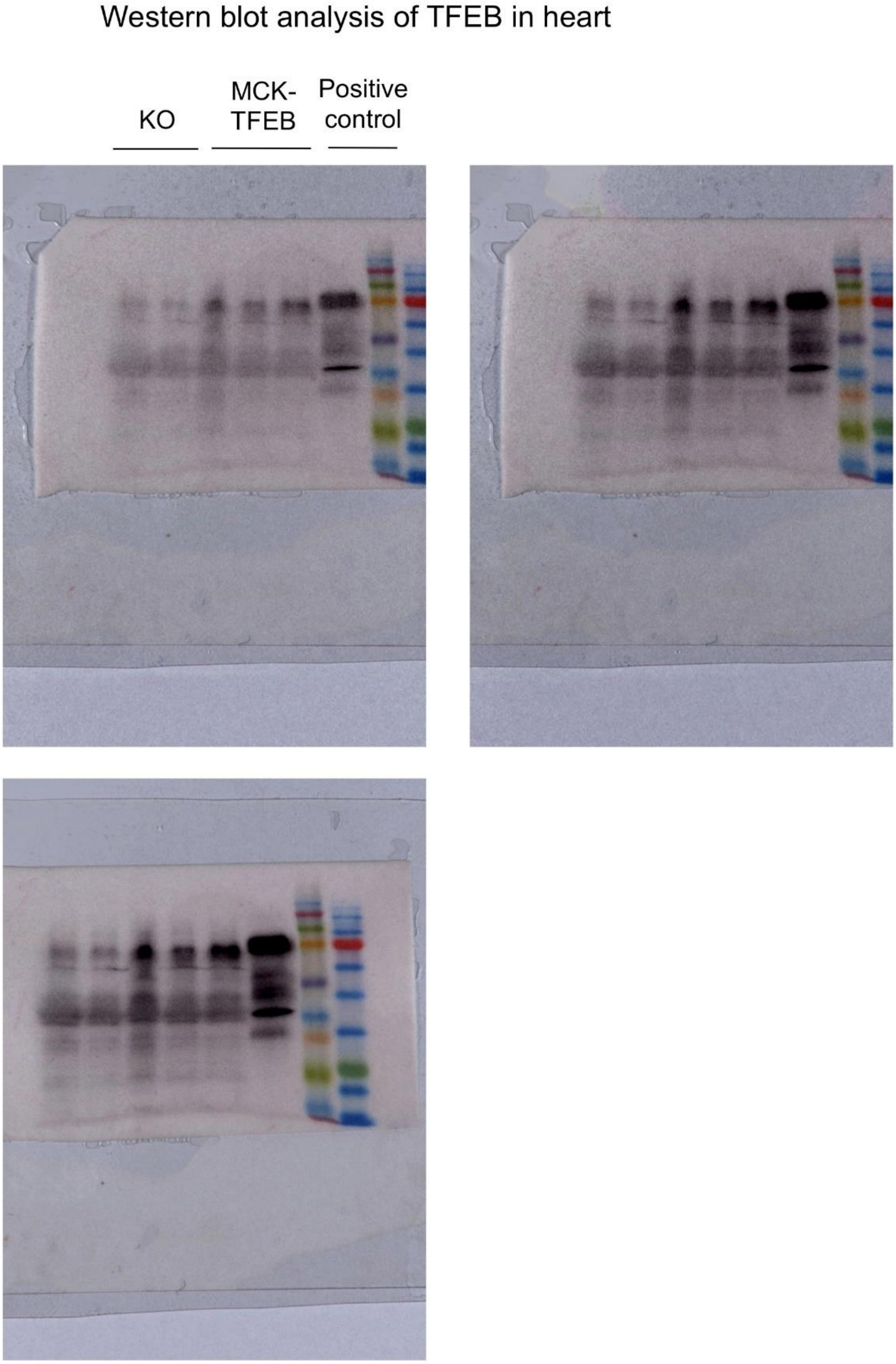

Figure S4. Original blots of anti 3xflag in heart at different exposures.

FIGURE S5

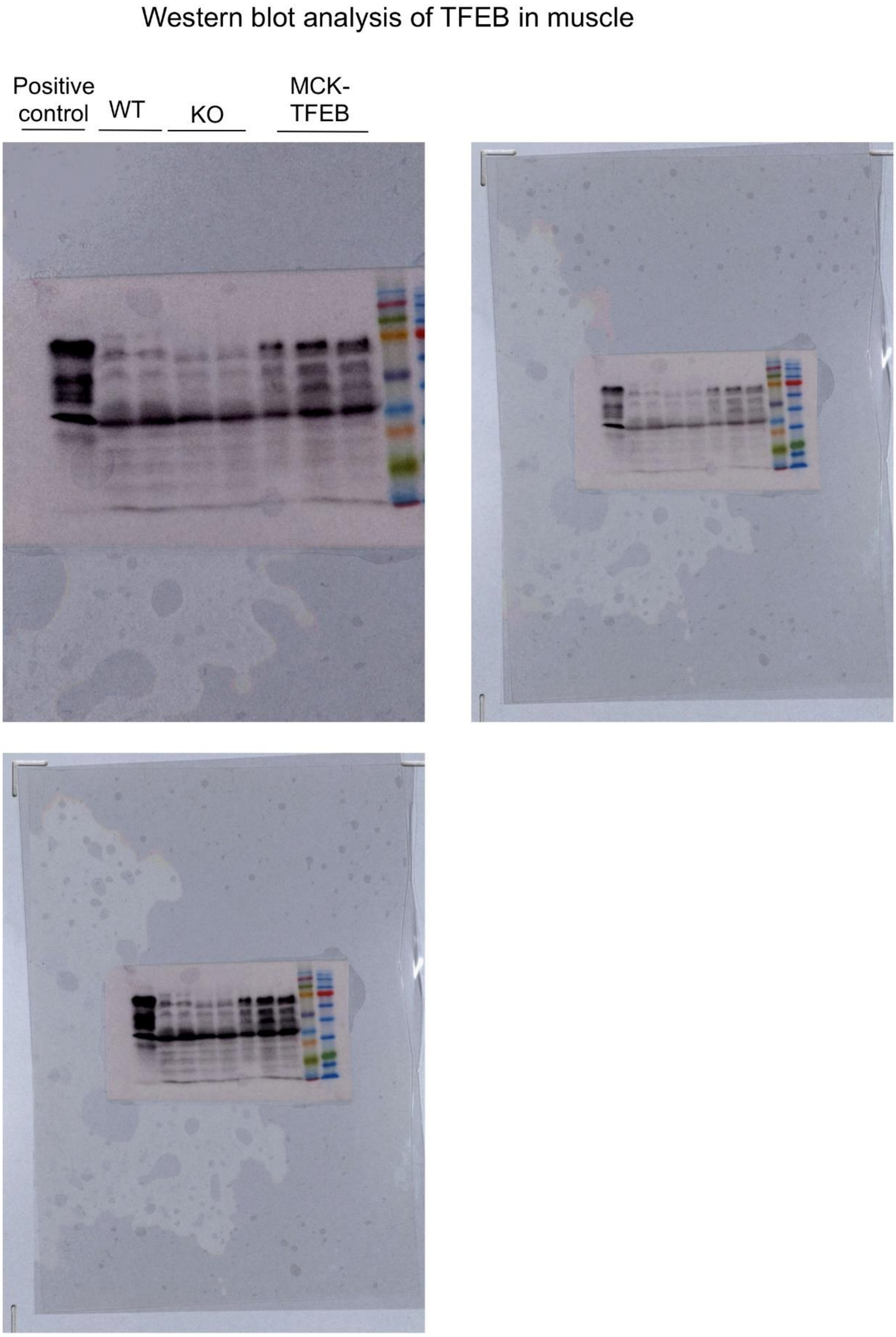

Figure S5. Original blots of anti 3xflag in gastrocnemius at different exposures.

Table S1

Sequences of primers used in real time PCR analyses

	**Forward primer**	**Reverse primer**
**Beclin**	AGCCTCTGAAACTGGACACG	ATGGCTCCTCTCCTGAGTTAG
**ATG 5**	CGAGATGTGTGGTTTGGACG	TCTCATAACCTTCTGAAAGTGCT
**ATP6V1H**	GTTGCTGCTCACGATGTTGGAG	TGTAGCGAACCTGCTGGTCTTC
**Atrogin**	GCAAACACTGCCACATTCTCTC	CTTGAGGGGAAAGTGAGACG
**TFEB**	GCAGAAGAAAGACAATCACAACC	GCCTTGGGGATCAGCATT
**GAPDH**	CACCATCTTCCAGGAGCGAG	CCTTCTCCATGGTGGTGAAGAC
**BGH**	TCTAGTTGCCAGCCATCTGTTGT	TGGGAGTGGCACCTTCCA
